# A Comprehensive Single Institutional Review of 2 Years in a Designated Fast-Track Sarcoma Diagnostic Clinic Linked with a Sarcoma Specialist Advisory Group: Meeting the Target but Failing the Task?

**DOI:** 10.1155/2016/6032606

**Published:** 2016-06-02

**Authors:** Zoltan Szucs, Dochka Davidson, Han Hsi Wong, Gail Horan, Philip W. P. Bearcroft, Ian Grant, Robert Grimer, Melanie A. Hopper, Helen Hatcher, Helena Earl

**Affiliations:** ^1^Cambridge University Hospitals NHS Foundation Trust, Cambridge Biomedical Campus, Hills Road, Cambridge CB2 0QQ, UK; ^2^The Royal Orthopaedic Hospital NHS Foundation Trust and Sarcoma SAG, Bristol Road, South Birmingham B31 2AP, UK; ^3^University of Cambridge Department of Oncology and NIHR Cambridge Biomedical Research Centre, Box 277, Hills Road, Cambridge CB2 0QQ, UK

## Abstract

*Background*. National guidelines prompted the implementation of a designated two-week wait referral pathway to facilitate the early diagnosis of sarcomas, to improve treatment outcomes.* Methods*. Patients referred to the Cambridge Sarcoma Diagnostic Clinic between January 2013 and December 2014 were identified through the electronic appointments system. Information was retrospectively retrieved about patient characteristics and details of the diagnostic pathway.* Results*. 17.3% of patients referred (69/397) were diagnosed with a malignancy. Of these, 59.3% (41/69) had primary sarcomas, 17.4% (12/69) had metastatic cancer, and 23.2% (16/69) had a different primary malignancy. 15% of the 41 sarcomas were <5 cm, 34% in the 5–10 cm range, and 51% >10 cm. Sarcomas diagnosed through this clinic represented 13% (41/315) of sarcomas managed at the centre during the same 2 years.* Conclusion*. While we achieved the target of 10% (41/397) sarcoma diagnosis rate in the rapid access clinic, only 15% of these were <5 cm better prognosis lesions. This calls into question the “real world” impact of such diagnostic clinics on early diagnosis of sarcomas. In order to enhance generic cancer diagnostic skills, training in these diagnostic clinics could be usefully integrated into national training curricula for both surgical and nonsurgical oncologists.

## 1. Introduction

Approximately 2,700 bone and soft tissue sarcomas are diagnosed each year in the UK, accounting for 1% of all cancer diagnoses [1]. In 2000 the first comprehensive national plan for investment and reform for cancer commissioned by the government [2] had set out ambitious plans to cut waiting times for diagnosis and treatment of all cancer types. In the white paper [2], Chapter 5 called for the immediate implementation of two-week maximum wait for an urgent outpatient appointment for all suspected cancers.

Due to the comparative rarity, a diagnosis of sarcoma is less commonly included in the differential by primary care physicians. National guidelines [3, 4] prompted urgent two-week wait (2ww) referrals to tertiary diagnostic centres for patients with a soft tissue mass with any one of the following features: (i) size > 5 cm; (ii) increasing in size; (iii) being deep to deep fascia; (iv) being painful; (v) being recurrent after previous excision. Patients with abnormal radiographic findings suspicious for bone tumours were also to be referred under the same 2ww rule.

The Improving Outcomes Guidance for Sarcoma [5] states that “only one in ten referrals of ‘suspicious lumps' will be a sarcoma.” We performed an in-depth comprehensive analysis of our diagnostic practice in order to determine its impact on the early diagnosis of sarcoma.

## 2. Methods

All patients referred under the 2ww rule to the Cambridge Sarcoma Diagnostic Clinic of the Cambridge University Hospitals NHS Foundation Trust (CUHFT) between 1 January 2013 and 31 December 2014 were identified through the electronic appointments system. The 2ww clinic with referral proformas had been developed within the Anglia Cancer Network and started in January 2011. These were available to all general practitioners (GPs) in the catchment area. The 2-year period detailed in this paper therefore represents the 2ww pathways in an established phase. Referrals were made for any soft tissue mass with one or more of the following established characteristics: larger than 5 cm, painful, increasing in size, deep to deep fascia, and recurring after previous excision or for any bone lesions with radiological suspicion of a primary bone tumour.

Each patient referred to our tertiary sarcoma centre had a clinical review and an individualized diagnostic pathway established. The outcomes of diagnostic investigations were discussed in the local Sarcoma Multidisciplinary Team Meeting (MDT) of CUHFT. All cases deemed suspicious for soft tissue or bone sarcoma were referred for further discussion in the designated site specific MDT of the Birmingham Sarcoma Specialist Advisory Group (suspected bone sarcomas and soft tissue sarcomas occurring in extremities to the Royal Orthopaedic Hospital NHS Foundation Trust and all other suspected soft tissue sarcomas to the University Hospitals Birmingham NHS Foundation Trust). The supraregional Birmingham Centres provided the surgical expertise of safe tissue sampling in highly suspicious cases for sarcoma and also the operative management once a sarcoma diagnosis was established (chemotherapy and radiotherapy as needed was delivered in the designated Sarcoma Unit of CUHFT).

Once the patients were identified a database was created retrospectively and the following data items were retrieved (using paper and electronic patient records): patient demographics, anatomical site and radiological size of the mass, imaging investigations leading to diagnosis, method of obtaining a diagnosis (radiological, histology, and clinical), final diagnosis, and geographic site of diagnosis (local versus supraregional surgical sarcoma centre).

## 3. Results

397 patients referred under the 2ww were reviewed in the sarcoma fast-track diagnostic clinic of CUHFT as part of the Cambridge Cancer Centre during the study period—188 patients in 2013 and 209 in 2014.

The mean age of patients was 56.1 years (range 16–93) with a female : male ratio of 1.04 : 1. The most frequent anatomical location of the suspected sarcoma was in the thighs with 66 (16.6%) cases, followed by the chest wall (33 cases, 8.3%) and the shoulders (32 cases, 8%) (see Supplementary Table 1 in Supplementary Material available online at http://dx.doi.org/10.1155/2016/6032606). In 338/397 (85.1%) patients the diagnosis was established locally in Cambridge and in 59/397 (14.9%) patients this was achieved at the Sarcoma Specialist Advisory Group/MDT in Birmingham. The final diagnosis was supported by histology results in 208/397 patients (52.4%). In 5 patients the diagnosis was based solely on clinical grounds. In 178/397 (44.8%) patients the final confirmatory diagnosis was based on the results of imaging studies (Table 1).

A wide range of imaging tools and combinations thereof were employed in order to achieve a diagnosis. 337/397 (84.9%) patients had objectively measurable lesions on radiological examination; the average size of all lesions was 58.3 mm (range 4–250). 239/397 (60.2%) patients had an US and 287/397 (72.3%) an MRI, on their own or as part of multiple imaging (Table 2).

192/397 (48.4%) patients were diagnosed with a benign tumour. The most frequently diagnosed benign tumours were lipogenic in nature (124), followed in order of frequency by neural lesions (21), vascular/lymphatic tumours (14), fibromatous growth (12), and bone tumours (9) (Table 3).

7.8% of patients (31/397) had an inflammatory or infective condition of some sort. 19 patients presented with a lesion secondary to trauma and 14 patients were diagnosed with cysts. Further 26 patients presented with “other” benign, nontumourous conditions, including fat necrosis (8), bursa (5), and endometriosis (4). No abnormality of any sort was detected in 14 patients, while 28 patients had normal anatomical variations/fatty lumpiness that resulted in their “lumps or bumps” (Supplementary Table 2).

Four patients with a mean age of 50.2 years (range 37–61) were diagnosed with a borderline tumour of 33 mm (range 18–45) average size. Pathologies included a fibromyxoid tumour, a giant cell tumour of tendon sheath, and two cases of pigmented villonodular synovitis of the joint.

69/397 (17.4%) patients were diagnosed with cancer, more specifically 41/397 (10.3%) with primary soft tissue or bone sarcomas, 16/397 (4%) with other primary malignancies, and 12/397 (3%) with metastatic cancers. Of patients diagnosed with malignancy bone or soft tissue sarcoma was the most common diagnosis: 41/69 (59.4%) (Table 4). The average age of patients diagnosed with a bone (4) or soft tissue (35) sarcoma was 59.7 years, the mean size of sarcomas was 106.3 mm. The highest prevalence of detected sarcomas was in the thigh (14), followed by retroperitoneum (4), pelvis (3), and chest wall locations (3). The mean size of the four diagnosed bone sarcomas was 98.5 (44–140) mm, while that of soft tissue sarcomas was 110.21 (10–240) mm. At presentation 6/41 (14.6%) sarcomas were smaller than 5 cm and 14/41 (34.1%) sarcomas were in the 5–10 cm range with the remaining 21/41 (51.2%) larger than 10 cm. The 4 retroperitoneal sarcomas (3 liposarcomas and 1 sarcoma with rhabdomyosarcomatous differentiation) were all larger than 10 cm. Excluding the retroperitoneal sarcomas and the two GISTs, the mean size of extremity and trunk wall soft tissue sarcomas was 102.62 mm.

Liposarcomas (mainly atypical lipomatous tumours) were the most frequently diagnosed sarcomas. The eight atypical lipomatous tumours mainly affected the thighs (6) with one case arising in the buttock and another one in the retroperitoneum. 20 sarcomas were of high grade (grade 3) at diagnosis. Seven patients had distant spread of disease at presentation and a further 3 developed metastatic disease within 1 year of primary diagnosis (Table 4). 29 patients were treated with a radical curative intent, while in 10 cases a palliative approach was followed from the diagnosis (6 patients being unsuitable for any active treatment). Eight sarcoma patients have died since the diagnosis (Table 5).

In the two years reviewed here, a total of 314 patients (151 in 2013 and 163 in 2014) were diagnosed with sarcoma and referred to the sarcoma team at CUHFT. Therefore only 41/314 (13%) of sarcoma patients were referred through the fast-track diagnostic clinic.

In addition to the diagnosed sarcomas, 12/69 (17.4%) had soft tissue lumps or bony abnormalities which were shown on biopsy to be metastatic cancer from a different primary source (Table 6) and 16/69 (23.2%) were diagnosed with another primary malignancy. Of 28 nonsarcomatous cancers (primary and metastatic) haematological malignancies accounted for 50% (14/28) with 8 having lymphomas, 5 having plasmacytomas, and 1 having multiple myeloma (Table 6). In addition, one patient was diagnosed with a primary Merkel cell skin tumour and a further patient with an adenocarcinoma within a vestigial urachal tract below the umbilicus and 12 metastatic cancers were diagnosed and the histological diagnoses are shown in Table 6.

## 4. Discussion

397 patients referred under the 2ww rule were reviewed in the sarcoma fast-track clinic of CUHFT. Approximately every sixth (17.4%) patient coming through the 2ww fast-track clinic was diagnosed with cancer. 4.5% were diagnosed with another primary malignancy and 3% with metastatic cancers. Every tenth patient (9.82%) was diagnosed with a primary soft tissue or bone sarcoma. These figures are similar to recently published 2ww outcome reviews, including the more detailed work of Barwick et al. [6]. The Improving Outcomes Guidance for Sarcoma states that “only one in ten referrals of suspicious lumps will be a sarcoma,” and our practice confirmed this figure.

The final diagnosis was obtained by histological sampling in more than half (52.4%) of the patients reviewed, reflecting a doubt about the nature of the lesions after radiological imaging. This data is consistent with appropriate referrals being made by primary care physicians.

Only 3.84% of locally established final diagnosis was sarcoma, while almost half (44.06%) of supraregional referrals yielded a sarcoma diagnosis. These figures clearly demonstrate that the local MDT was efficient in channeling suspected sarcoma patients with a high or moderate suspicion of sarcoma to the appropriate supraregional service for safe tissue sampling. In addition, in all patients with a locally established sarcoma diagnosis the biopsy was performed in agreement with the supraregional centre.

It has long been recognised that any soft tissue mass larger than 5 cm holds a significantly higher chance of being malignant [7–9]. 5- and 10-year survival rates dramatically drop for nonmetastatic sarcomas larger than 5 cm at presentation, with significant decrease in survival for every incremental 5 cm increase in size [10]. In a more recent series [11] the average size of sarcomas of those referred under the 2ww rule was actually slightly higher (10.1 cm versus 9.3 cm) than for those referred routinely (*p* = 0.28). In a comprehensive review of trends in presentation of bone and soft tissue sarcomas [12], the size of bone sarcomas had not changed over time but there had been a slight decrease in the size of soft tissue sarcomas (10.3 cm before 2000 versus 9.6 cm after 2000, *p* = 0.03). In our own series the mean size of the 41 sarcomas diagnosed was 10.6 cm. At presentation only 6 sarcomas were smaller than 5 cm and 14 sarcomas were in the 5–10 cm range. Half (21/39) of the sarcomas were larger than 10 cm. 22 sarcomas were high grade at diagnosis, which is an unfavourable prognostic factor of its own. Seven patients had distant spread of disease at presentation, further 3 developing metastatic disease within 1 year of primary diagnosis (Table 4). In 10/39 sarcoma cases the therapeutic approach was palliative from the very beginning, 6 patients being unsuitable for any active treatment because of poor performance status and comorbidities. While only two and a half years have elapsed since the very beginning of the study entry, 8 sarcoma patients have already died since their diagnosis.

The overall size of 10.6 cm is more than double the size indicated in the national guidelines previously quoted. Our results contribute to the gradually increasing body of scepticism regarding the effectiveness of the easy-access 2ww fast-track diagnostics to meet their primary aim of diagnosing sarcomas early [6, 11, 13, 14]. There is concern that as a consequence of running such continuously expanding clinics, trained personnel and financial resources are removed from the highly specialised care of patients already diagnosed with sarcoma.

While ultrasonography was suggested to be less reliable than magnetic resonance imaging for further investigation of soft tissue masses [15], it was found to be an effective diagnostic triage tool for the evaluation of soft tissue mass cases referred from primary care [16]. Imaging triage of soft tissue masses proved to be safe and reliable in detecting benign lumps while increasing the proportion of indeterminate and malignant lesions cases referred to specialist centres [17]. In our own series the proportion of benign tumours was high; almost half (48.4%) of the patients were diagnosed with a benign growth. Nevertheless, in 64 cases (Table 3) ultrasound on its own was a useful imaging tool to lead to a final diagnosis of a benign lesion. Our study supports recommendations [11, 14] that patients with suspicious lumps should be investigated by ultrasound prior to a 2ww referral. Our belief and advice is that this should be done by a radiologist with expertise in musculoskeletal radiology.

The most recent 2015 NICE guidelines [18] have reviewed the lack of evidence with regard to reliable alarm symptoms and signs in the early diagnosis of sarcomas. No specific symptoms have exceeded the above 3% positive predictive value threshold in the diagnosis of either bone or soft tissue sarcomas, and therefore none of the five classical alarming sarcoma symptoms were included as a 2ww referral criteria. Recommendations have been made for suspected cancer pathway referral from primary care in the case of a suspicious radiographic result (bone sarcomas) or ultrasound result (soft tissue sarcomas) [18]. These guidelines by default render much more freedom to the GPs in evaluating sarcoma alarm symptoms in the community and decrease the burden of referrals toward tertiary centres. It can be argued that these new guidelines also hold an inherent risk of leading to missed sarcoma diagnosis due to the relative lack of exposure to musculoskeletal radiology of community ultrasonographers. We also need to ensure that the awareness of the classical sarcoma alarm symptom “quintet” does not get forgotten by primary care physicians.

The Independent Cancer Task Force in their next 5-year vision paper listed a national ambition of achieving earlier diagnosis of cancer as part of their six key priorities [19]. They propose a shift towards faster and less restrictive investigative testing and giving GPs direct access to key investigative tests. The task force encourages the testing of new models, which could reduce the burden and expectation on GPs.

The large group of sarcomas encompassing more than 80 different histotypes are varied in their biological behaviour and anatomical location of origin affecting the whole body (often mimicking the symptoms of a wide variety of other solid tumours), thus rendering the early diagnosis of this specific tumour type potentially difficult. As reflected in this study, we believe that, based on the complex investigational pathways and spectra of established diagnoses in a sarcoma diagnostic practice, these services strongly enhance the general diagnostic skills of the oncologists involved. We suggest that each cancer centre involved in the teaching of the future generation of both surgical and nonsurgical oncologists may consider setting up such practices regardless of the level of their involvement in the management of sarcomas.

## 5. Conclusion

We have achieved the targets outlined in the Improving Outcomes Guidance for Sarcoma document with a 10% diagnosis rate of sarcoma. However, our results suggest that there is little evidence that the two-week wait clinic is achieving the task of diagnosing sarcomas at an earlier and more treatable stage. There is genuine concern that by running such continuously expanding clinics, trained personnel and financial resources are removed from the highly specialised care of patients already diagnosed with sarcoma. Sarcoma diagnostic clinics yet still offer an excellent template of enhancing diagnostic skills of all cancer types and thus involvement in its work could be usefully integrated into oncology training curricula.

A consensus of sarcoma experts on national and international levels is needed to provide clear guidance in regard to improving services nationally for a genuinely early diagnosis of bone and soft tissue sarcomas.

## Supplementary Material

Supplementary Table 1 Anatomical distribution of referred lesions.
Supplementary Table 2 Benign Lesions of Non-Tumourous Nature.

## Figures and Tables

**Table 1:** Diagnostic pathways and results.

	Method of diagnosis	BT	T	I	BE	P	M	S	SM	Total
Local	Clinical	1	1	1	2					5
	Excisional biopsy, plastic surgeon	84		5	12	1		4		106
	Histology, colorectal surgeon						1			1
	Histology, neurosurgeon		1					1		2
	Histology, orthopaedics	1							2	3
	Biopsy (radiology guided)	10		5	4	12	7	7		45
	BMT					2				2
	Radiological	74	16	20	60		3	1		173
	Total	170	18	31	78	13	11	15	2	338
	*% (of all local)*	50.29	5.32	9.17	23.07	3.84	3.25	4.43	0.59	100

Supraregional	Histology, Bham	14	1			2		21	2	43
	Histology, Pworth	1				1	1	3		5
	Radiological, Bham	6			4			2		10
	Radiological, RMH	1								1
	Total	22	1		4	3	1	26	2	59
	*% (of all supraregional)*	7.28	1.69	0	6.77	5.08	1.69	44.06	1.69	100

All	Total	192	19	31	82	16	12	41	4	397
	*% (of all)*	48.36	4.78	7.80	20.65	4.03	3.02	10.32	0.75	100

BT: benign tumours, T: traumatic lesion, I: inflammatory/infective lesions, BE: benign lesions of nontumorous nature or no abnormality detected, P: primary cancer, M: metastatic cancer, S: sarcoma, SM: semimalignant tumour, BMT: bone marrow trephine, Bham: Royal Orthopaedic Hospital Birmingham or the Midland Abdominal and Retroperitoneal Sarcoma Unit (MARSU), Pworth: Papworth Hospital, and RMH: The Royal Marsden Hospital.

**Table 2:** Pattern of radiological investigations and malignancy detection rates.

Imaging modality	All	Measurable	Average size (range) mm	Metastatic cancer	Primary cancer	Sarcoma
US	64	54	33.55 (4–130)			
MRI	61	54	48.33 (9–150)			1
CT	19	16	108.43 (24–200)	4	3	10
X-ray	6	0				
US MRI	113	106	53.79 (8–154)			1
US CT	9	8	51.37 (12–150)	1	1	1
US X-ray	3	2	25 (10–40)			
MRI CT	33	31	83.26 (20–250)	2	3	10
MRI X-ray	19	7	48.71 (18–110)			
CT X-ray	2	0				
CT NM	1	1	40			
X-ray NM	1	0				
US MRI CT	46	46	76.27 (18–222)	3	6	14
US MRI X-ray	4	2	35.5 (6–65)			
MRI CT X-ray	6	6	67.50 (40–150)	1		2
MRI CT NM	3	3	86 (38–140)		1	2
MRI X-ray NM	1	0				
US MRI X-ray CT NM	1	1	40		1	
Did not attend US	1	0				
No imaging required	4	0				
*Total*	*397*	*337*	*58.26 (4–250)*	*12*	*16*	*41*

US: ultrasound, MRI: magnetic resonance imaging, CT: computed tomography, and NM: bone isotope scan.

**Table 3:** Diagnostic distribution of benign tumours.

*Lipogenic tumours*	
Angiolipoma	7
Lipoma (simple)	86
Lipoma (intra-articular)	1
Lipoma (intramuscular)	12
Lipoma (multiple simple)	6
Lipoma (spindle cell)	8
Lipoma with fat necrosis	1
Lipomatosis	1
Angiomyolipoma	1
Fibrolipoma	1
*Bone tumours*	
Benign giant cell tumour	1
Cystic medullary bone tumour	1
Enchondroma	3
Exostosis	1
Intraosseous ganglion	1
Osteochondroma	2
*Neural tumours*	
Neural tumour	1
Neurofibroma	1
Neuroma benign	1
Peripheral nerve sheath tumour	4
Schwannoma	14
*Vascular/lymphatic tumours *	
Angiomyofibroblastoma	1
Arteriovenous malformation	2
Glomus tumour	2
Haemangioma	6
Lymphangioma	1
Masson's papillary endothelial hyperplasia	1
Venous malformation	1
*Fibromatous tumours*	
Benign fibroelastoma	1
Desmoid-type fibromatosis	7
Fibroepithelial polyp	1
Fibrous dysplasia	1
Fibrovascular tissue	1
Plantar fibromatosis	1
*Other*	
Ganglion	4
Granular cell tumour	1
Myxoma	5
Pilomatrixoma	1
Sarcoid, foreign body	1
Spiradenoma	1

**Table 4:** Diagnosed sarcomas according to tumour type, grade, and metastatic nature.

Diagnosis (number)	Age (range)	Size mm (range)	G?	G1	G2	G3	M(P)	M(E)
Chondrosarcoma (1)	60	90		1				
Ewing's sarcoma (3)	20.33 (17–22)	106.66 (80–140)				3	2	
Undifferentiated (pleomorphic) sarcoma (6)	69.33 (40–89)	80.33 (40–130)				6	3	1
Leiomyosarcoma (3)	57.66 (30–70)	80 (45–150)		1	1	1		
Liposarcoma (13)	66.61 (34–93)	137.38 (64–240)	3	8	2			
Myxoid liposarcoma (1)	69	160				1		
Myofibroblastic sarcoma (1)	68	44				1		
Myxoid fibrosarcoma (4)	64 (52–81)	80.25 (60–100)			2	2		2
Osteoblastic osteosarcoma (1)	33	110				1	1	
PEComa (1)	58	74				1		
Sarcoma with rhabdomyosarcomatous differentiation (1)	60	180				1	1	
Spindle cell sarcoma (3)	65 (26–85)	108.33 (10–220)		1		2		
Synovial sarcoma (1)	53	40				1		
Gastrointestinal stromal tumour (2)	58.5 (54–63)	150 (100–200)				2	1	

G?: radiological diagnosis only and therefore grade is unknown, G1: grade 1, G2: grade 2, G3: grade 3, M(P): metastatic at presentation, and M(E): early (<1 year) metastatic relapse.

**Table 5:** Therapeutic approach and management of the sarcoma patients diagnosed in the 2ww clinic.

	Curative			Palliative			Died
	Surgery	Surgery + Tx	Chemo/Rad	Rad	Chemo	BSC	
Chondrosarcoma (1)	1						
Ewing's sarcoma (3)		1	2				
Undifferentiated (pleomorphic) sarcoma (6)	1	2	1	2			2
Leiomyosarcoma (3)	3						
Liposarcoma (13)	6	2				5	3
Myxoid liposarcoma (1)		1					
Myofibroblastic sarcoma (1)				1			
Myxoid fibrosarcoma (4)	1	3					
Osteoblastic osteosarcoma (1)		1					1
PEComa (1)		1					
Sarcoma with rhabdomyosarcomatous differentiation (1)					1		1
Spindle cell sarcoma (3)	1	1				1	1
Synovial sarcoma (1)		1					
GIST (2)			1		1		

Tx: perioperative neoadjuvant/adjuvant radio- and/or chemotherapy, Chemo: chemotherapy, Rad: radiotherapy, BSC: best supportive care, PEComa: perivascular epithelioid cell tumour, and GIST: gastrointestinal stromal tumour.

**Table 6:** Nonsarcomatous cancer diagnosis.

*Metastatic cancer*	
Breast cancer	2
Colorectal cancer	1
Lung cancer	3
Renal cell carcinoma	2
Transitional cell carcinoma	1
Paraganglioma	1
Squamous cell carcinoma (skin)	1
Nonseminomatous germ cell tumour	1
*Primary cancer*	
Classical Hodgkin's lymphoma	1
Cutaneous marginal zone lymphoma	1
Follicular lymphoma	3
High grade B-cell lymphoma	1
Anaplastic large cell lymphoma	1
Mantle cell lymphoma	1
Plasmacytoma	5
Myeloma	1
Merkel cell carcinoma	1
Urachal adenocarcinoma	1
